# Effect of Temperature Variation and Pre-Sustained Loading on the Bond between Basalt FRP Sheets and Concrete

**DOI:** 10.3390/ma13071530

**Published:** 2020-03-26

**Authors:** Jun He, Zhongyu Lu, Sirong Tan, Tamon Ueda, Yunfeng Pan, Jianhe Xie, Guijun Xian

**Affiliations:** 1 School of Civil Engineering, Chang’an University, Xi’an 710061, China; hejun@chd.edu.cn; 2 School of Civil and Transportation Engineering, Guangdong University of Technology, Guangzhou 510006, China; luzy@gdut.edu.cn (Z.L.); jhxie@gdut.edu.cn (J.X.); 3 School of Civil Engineering and Architecture, Guangxi University for Nationalities, Guangxi Zhuang Autonomous Region 530000, China; 20195019@gxun.edu.cn; 4 Guangdong Province Key Laboratory of Durability for Marine Civil Engineering, College of Civil and Transportation Engineering, Shenzhen University, Shenzhen 518060, China; ueda@szu.edu.cn; 5 School of Civil Engineering and Architecture, Zhejiang Sci-Tech University, Hangzhou, 310018, China; yf.poon@foxmail.com; 6 School of Civil Engineering, Harbin Institute of Technology, Harbin 150090, China

**Keywords:** basalt fiber-reinforced polymer (BFRP) sheet, concrete, interface, pre-sustained loading, temperature variation

## Abstract

The coupled effects of temperature variation and pre-sustained loading on the bond between basalt fiber reinforced polymer (BFRP) sheets and a concrete substrate were studied. Single lap-shear test specimens were exposed to temperatures of 15, 30, 40, 50, and 60 °C for 3 h with pre-sustained loading at 35% of the ultimate load capacity (*F*_u_). Compared with the case of 15 °C, the interfacial fracture energy of the specimens at 30 and 40 °C increased by 46% and 11%, respectively, whereas those reduced by 73% and 77% at 50 and 60 °C, respectively. The coupled effects of temperature and pre-sustained loading on the effective bond length are insignificant for the specimens at both 15 and 30 °C and the effective bond length increased to 300 mm when the temperature exceeded 40 °C. The failure crack still occurred in the concrete substrate at the temperatures of 15 and 30 °C, and changed to the debonding of the adhesive layer from the concrete substrate at the temperature above 30 °C.

## 1. Introduction

Externally bonded fiber-reinforced polymer (FRP) sheets or plates have become popular for strengthening reinforced concrete structures in recent decades. The effectiveness of this technique relies on the integrity of the bond between the FRP sheet/plate and concrete [1,2]. Hitherto, numerous studies were conducted to investigate the short-term behavior of the FRP-to-concrete bond, both numerically and experimentally [3,4,5,6]. The harsh service environment of sustained loading, elevated service temperature (EST), and their combination deteriorates the FRP-to-concrete bond [7,8,9,10,11,12]. EST denotes the elevation of the matrix material and the bonding adhesive slightly above the glass transition temperature (*T*_g_) [13]. FRP composites exhibit a poor resistance to EST because organic polymers are used as the matrix materials. Above the *T*_g_, the elastic modulus of the matrix materials significantly drops and the material changes from a hard, glass-like material to a more rubber-like material [14]. The bonding adhesive softens quickly around the *T*_g_ and the sustained loading may deteriorate the bond between the adhesive layer and concrete. Studies of the influences of temperature variation, sustained loading, and the combination of both on the FRP-to-concrete bond are scarce. Therefore, although the externally bonded FRP technique is well established, it is essential for designers to understand how the FRP-to-concrete interface behaves under variable temperature conditions and sustained loading.

The temperature variation influences the epoxy and the FRP-to-concrete interfacial bond significantly. Leone et al. investigated the effects of temperature at 20, 50, 65, and 80 °C on the CFRP (carbon FRP)/GFRP (glass FRP) sheet-to-concrete by using a double-lap shear test [8]. The *T*_g_ of the epoxy resin is 55 °C. The ultimate load capacity of CFRP sheet-to-concrete increased in the temperature range from 20 to 50 °C (i.e., below *T*_g_). Compared to the case of 20 °C, 50 °C increased the ultimate load capacity by 24%. However, at 80 °C, which is above the *T*_g_, the ultimate load capacity was reduced by 10%. Ferrier et al. adopted double-lap shear tests to investigate the bond performance of CFRP sheet/plate-concrete exposed to temperatures in the range of −40 to 120 °C [15]. In the case of *T*_g_ = 76 °C for the epoxy resin, the EST at 80 °C reduced the ultimate load capacity by 70% for the CFRP sheet-concrete. In the case of *T*_g_ = 90 °C for the epoxy adhesive, the EST at 100 °C reduced the ultimate load capacity by 31% for the CFRP plate-concrete. Adelzadeh investigated the CFRP plate-concrete at room temperature, 60, 80, 105, 150, and 200 °C by a single-lap shear test [16]. The *T*_g_ of the epoxy adhesive (Sikadur30) is 60 °C. A 50% loss of strength for CFRP plate-concrete bonds was observed at a temperature 40 °C above the *T*_g_ of the adhesive. The reduction in the mechanical properties of the adhesive at EST is important for the strengthened structures, primarily in relation to the bond performance. The transformation in the failure mode from concrete cohesive failure to adhesive-concrete interfacial debonding failure causes the degradation of the ultimate load capacity. Dai et al. developed a theoretical nonlinear local bond-slip model for FRP laminates externally bonded to concrete at elevated temperature [9]. The prediction showed that the ultimate load capacity reduced to zero when the temperature in the adhesive layer was twice that of *T*_g_ (°C). This leads to the deterioration of the physical bonding between epoxy and concrete, which is formed by strong hydrogen bonds between the epoxy and SiO2 (concrete), involving highly polar aliphatic hydroxyl and ether groups in epoxy adhesive chains [17]. The service temperature proximity to *T*_g_ results in an increased rate of physical aging, which in turn results in the evolution of material properties. Furthermore, service temperature denotes one below the *T*_g_ of the matrix material and the bonding adhesive. Physical aging at the service temperature was identified as the reason for the increase in *T*_g_ of the resin [18]. Temperatures close to and above the *T*_g_ the of adhesive reduced elastic modulus of the adhesive [19]. 

Sustained loading significantly influences the bond strength of FRP-to-concrete. Jeong et al. investigated the effects of curing time, service temperature, and sustained loading duration on the mechanical behavior of CFRP sheet/concrete-bonded connections by using a single-lap shear test [11]. The sustained loading increases the ultimate load capacity of CFRP sheet-to-concrete by 23% after 31 days at 23 °C. The positive effects of sustained loading on the interfacial bond decreases with duration, and level of sustained loading. Jia et al. investigated the effects of the indoor laboratory, outdoor, elevated temperature/dry, and freeze/thaw on glass sheet-concrete bonds with sustained loads through a three-point flexure test [20]. Twenty-five percent of the calculated nominal ultimate moment in ambient indoor temperature did not vary the bond strength of the glass fiber reinforced-polymer-concrete-bonded interfaces. Because of the different creep characteristics of the constituent materials, the duration time, and sustained loading may induce stress concentration at the interface and influence the stress transferred to the FRP. Zhou et al. investigated the coupling of the sustained load and moisture conditions on the concrete-epoxy interface bond [21]. A sandwiched concrete–epoxy beam was adopted and applied to a three-point bending test. The results of the three-point bending test show that the sustained loading with 30% of *F*_u_ decreases the interfacial fracture energy of concrete-epoxy interface by 41% after four weeks. Sustained loading increases the effective bond length. A pre-stressed FRP sheet bonded to a beam and a double lap shear test was conducted [22]. The pre-stress level was 20%–82% of the failure load. A 70–80 mm relaxation zone in the double-lap shear test was reported after 4.3 days, at 50% of the initial ultimate load capacity in the double-lap shear test. The effective bond length continued to increase with the duration of sustained loading in terms of the strengthening concrete beam with pre-stressed FRP sheets.

The above review clearly illustrates that both sustained loading and temperature variation are critical in the bond performance. At the service temperature (below *T*_g_), the temperature effects are insignificant in specimens subjected to sustained load [11]. The combined effect of the temperature and mechanical loads becomes important for large differences in the coefficient of thermal expansion with the increase in the temperature [12]. However, relatively few studies have been conducted on the FRP-concrete interface at variable temperatures under service loads. Hence, in this study, the combined effects of temperature variation and sustained loading on the interface behavior of FRP-concrete were investigated, together with the interfacial fracture energy, bond stress–slip relationship, and effective bond length. A two-stage prediction equation of fracture energy is proposed considering the coupled effects of temperature variation and sustained loading. Basalt fiber has better mechanical properties than E-glass fiber, and is more widely available and cheaper than carbon fiber. Basalt FRP is believed to be a good alternative in civil engineering; therefore, BFRP sheets were bonded to concrete blocks in order to study the bond behavior of the BFRP sheet-to-concrete system.

## 2. Experimental Procedure

### 2.1. Raw Materials

To perform the single-shear tests, 18 rectangular concrete specimens with dimensions of 150 × 150 × 350 mm^3^ were prepared. Mix proportions were based on ACI standard [23]. The mixture proportions by the weight of the constituents were as follows: cement (1.0), water (0.5), coarse aggregate (2.78), fine aggregate (1.50). Ordinary Portland cement (OPC) was used in present study. The detailed physical properties and chemical compositions of OPC were reported in [24]. The maximum size of fine aggregate and coarse aggregate size was 1.2 and 20 mm, respectively. Air-entraining agent was not added. All of concrete were mixed one time. Firstly, mortar specimens were cast and cured for 24 h prior to removing the form. After demolding, specimens were cured under moist conditions (90 %) in the laboratory for 28 days at temperature of 20 ± 3 °C. In addition, three cylindrical specimens with 100 mm in diameter and 200 mm in height were cast to measure the compressive strength of the concrete. The materials and curing condition was the same to the rectangular concrete specimen. The average cylinder concrete compressive strength and its standard deviation was reported to be 32.8 and 2.9 MPa, respectively [25].

The BFRP sheets used in this study were fabricated via the wet layup process. Unidirectional untwisted basalt fiber was provided by the Tuo Xin Aerospace Basalt Industrial Co., Ltd. (Chengdu, China). The properties of the basalt fiber were provided by the manufacturer. The weight of the basalt fiber sheets was 700 g/m^2^. The average diameter of the basalt fibers was 13.5 µm, and the density was 2.60 g/cm^3^. The tensile strength, elastic modulus, and elongation at break of the basalt fiber were 2.44 GPa, 80.45 GPa, and 3.06%, respectively.

Epoxy-R and primer were the two-component resin with the base and hardener mixed in the ratio of 2:1 by weight. The tensile test and thermal analysis were conducted for the primer and epoxy using DMA (Dynamic Thermomechanical Analysis), respectively. Each test had two specimens. In the tensile test, the geometry of specimens was type II according to the ASTM D638 [26] and cured one week before tension. In the DMA analysis, the curing conditions was similar to that of the BFRP-to-concrete specimens. *T_g_* was determined by DMA thermal analysis at the begin of the single lap shear test [27] and set as the onset of the storage modulus curve [26]. The tensile strength, elastic modulus of Epoxy-R was found to be 56.74, 3100 MPa, and its standard deviation were 3.21, 140 MPa, respectively, while the tensile strength, elastic modulus of the primer was 64.2 and 3300 MPa, and its standard deviation were 1.36 MPa, 210 GPa, respectively. The *T*_g_ of the primer and Epoxy-R were 48.2 and 53.7 °C, respectively. 

### 2.2. Test Setup and Instrumentation

**Figure 1:**
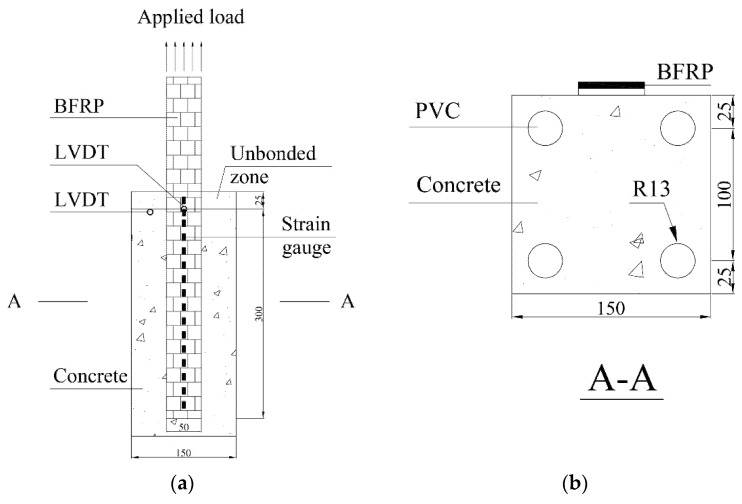
Details of bond specimens (all units in mm): (**a**) overall diagram; (**b**) section A-A.

The effective bond length was determined as follows [28]:

(1) $$L_{\mathrm{eff}}=\sqrt{\frac{E_{f}t_{f}}{\sqrt{f_{c}^{\prime }}}}$$

where *L*_eff_ is the effective bond length (mm); *E*_f_ is the elastic modulus of FRP (MPa); *f*_c_’ is the cylinder concrete compressive strength (MPa); *t*_f_ is the thickness of the FRP (mm). The effective bond length is 141 mm with *E*_f_ = 80.45 GPa, *f*_c_’ =32.8 MPa, and *t*_f_ =1.41 mm. It was reported that the EST increased the effective bond length [8]. When the environmental temperature reaches to *T*_g_, the effective bond length is more than 2 times of the control specimens with room temperature. Therefore, a bond length of 300 mm was adopted for all the FRP-concrete specimens in present study. The BFRP sheet was 50 mm wide and 1.41 mm thick. All of the concrete blocks were kept in the laboratory to cure for 28 days. The bonding surface of the concrete prism was ground by a disk sander to remove the thin mortar layer; followed by blown off of dust and any loose particles using compressed air. A layer of epoxy primer was applied on the surface and curing for 24 h before attaching the basalt-fiber sheet with the necessary saturation. The BFRP used by the basalt fiber and the Epoxy-R were prepared following wet up procedure. The BFRP–concrete specimens were cured for one week under the laboratory condition. All specimens of BFRP-to-concrete were tested within 3 weeks after the beginning of the test. Figure 1 shows the dimensions of the BFRP sheet-concrete specimens. The FRP axial strains were measured using 11 electrical resistance foil strain gauges along the centerline of the BFRP plate. The 11 electrical resistance foil strain gauges were spaced at 20 mm. The dimensions of the strain gauges are 7.4 × 4.9 mm^2^. Both strain gauges (BA 350-3AA) and adhesive (H61) were produced by Zhonghan Electronic Measuring Instruments Co., Ltd. H61 is adopted for adhesion between strain gauges and BFRP. The strain gauge and H61 are suitable at the range from −80 to 150 °C and from −30 to 150 °C, respectively. The instruction provided by manufacturer shows that H61 can be completely cured at the room temperature for two days. Two LVDTs (linear variable displacement transducer) were fixed at the BFRP plate and concrete substrate to measure the slip between BFRP plate and concrete substrate. One LVDT was positioned at the starting point of the bonding zone to measure the loaded end slip between the concrete and BFRP plate. The second LVDT was positioned on the concrete surface to measure the longitudinal displacement of the concrete.

**Figure 2:**
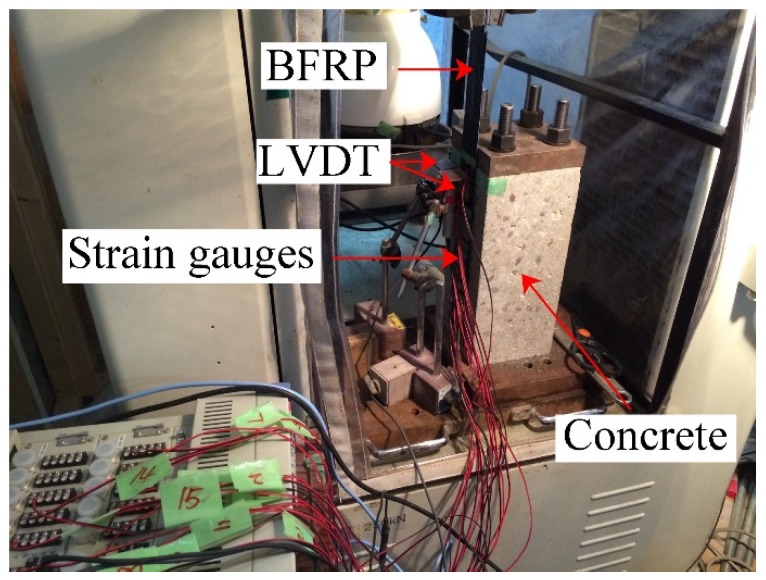
Detailed test setup of the single-lap shear test.

Figure 2 shows the details of the single-lap shear test. Three control specimens were tested to determine the ultimate load capacity (*F*_u_ = 26.3 kN). The Chen and Teng’s model, and Dai et al.’s model were adopted to confirm the bond strength [28,29]. It was determined to be 20.7 and 25.8 kN, respectively. A pair of aluminum sheets was used as an anchorage system, and the bonding tests were conducted on a universal testing machine (UTM) with a loading rate of 0.2 mm/min. Four screws were placed through the pre-set holes to affix the specimen on the test machine. The holes were formed using polyvinylchloride tube in the concrete specimens. The specimen was adjusted to make the BFRP parallel with the loading grip. 

Two types of tests are performed to study the effects of coupled temperature and sustained load on the bond behavior between BFRP systems and concrete: steady-state tests (STs) and transient test (TT). ST represents the BFRP-to-concrete system exposure to a short-term sustained load. The shear test to failure was applied in TT. The limitation value of the sustained stress for BFRP is not mentioned in ACI 440 [30]. According to the previous studies on the creep behavior of FRP-concrete, no creep bond failure was observed with the sustained level less than 50% of its ultimate load [23]. 35% of the ultimate load of BFRP sheet-to-concrete bond was adopted to simulate realistic worst-case loading conditions [31]. Thus, the sustained loading was chosen as 35% of the ultimate load capacity (9.2 kN) in present study. The specimens were first loaded to 9.2 kN at a loading rate of 0.2 mm/min, and the temperature was raised at a heating rate of 3 °C/min from the room temperature to the predetermined temperature after the load reached up to 35% of *F*_u_. The load was controlled by the universal testing machine, with the control precision within ± 0.2 kN. 

In the application of the FRP strengthening of concrete beams, the surface of FRP-to-concrete usually covers with a fire protection layer. The insulation layer can prevent flame spread and smoke generation, and in the meantime, ensuring that an adequate structural resistance is retained during a fire [9]. However, the heating transfer from the outer of the beam to the FRP-concrete interface. The temperature at the interface between FRP and concrete rises to hundreds of degrees Celsius under fire situation. The mechanical properties of the epoxy rapidly loss when the environment temperature is larger than *T*_g_. Thus, the temperatures were set to room temperature (approximately 15 °C), 30, 40, 50, and 60 °C. As previous discussed, *T*_g_ of the primer is 48.2 °C, and the load capacity of the interfacial bond rapidly losses after the environmental temperature above *T*_g_. Thus, the highest exposure temperature was set as 60 °C. The testing temperature was controlled by an electrically heated chamber at a rate of approximately 10 °C/min. The temperature at the surface of the BFRP laminate was measured in the experiment. A thermocouple was adopted to read the temperature. The thermocouple was placed at the surface of the BFRP, which is 150 mm from the free end. Fire-resistance of elements of the building construction before the collapse requires at least 128 min according to ISO 834 [32]. The heating involved by fire transfers to the bond interface of the BFRP-to-concrete. The BFRP-to-concrete can bear a load for three hours under the coupled of temperature and sustained load. Thus, three hours was applied in present study. The specimens were exposed to the predetermined temperature with sustained loading for 3 h. Consecutively, the load was released to zero at the rate of 0.2 mm/min. A shear test was performed immediately at the predetermined temperature with a loading rate of 0.2 mm/min. In addition, a set of specimens was tested at 15 °C without sustained loading in the same condition for comparison.

It is should be noted that the experimental condition is unloading to zero after sustained loading for 3 h and then loading from zero to *f*_u_ during the single lap-shear test. This is similar to other research on the effect of sustained loading. In fact, this investigates the effect of the pre-sustained loading before the single lap-shear test and temperature variation on the bond behaviors on BFRP-to-concrete joints. Therefore, it is more accurate to describe this experimental condition using the phrases of pre-sustained loading.

## 3. Results and Discussion

### 3.1. Failure Mode of BFRP–Concrete Bond

**Figure 3:**
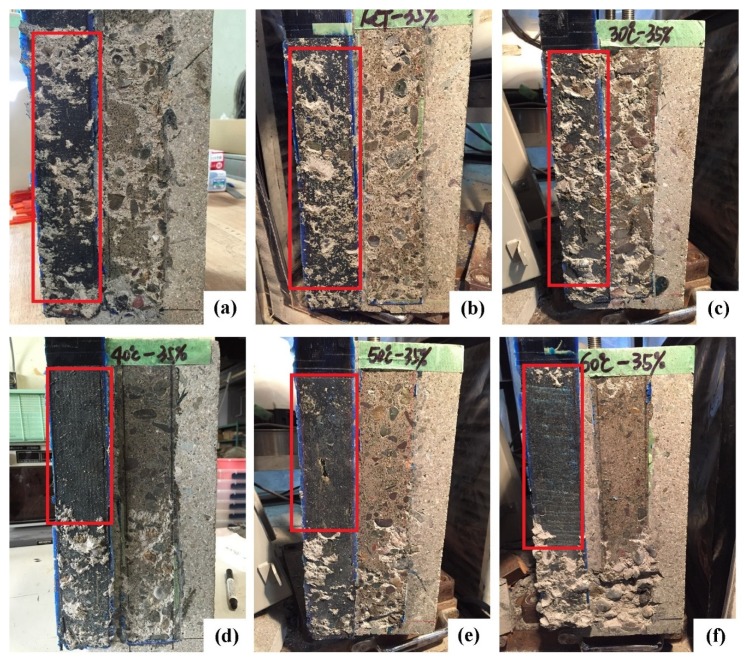
Comparison of failure surfaces at various exposure conditions: (**a**) control; (**b**) 15 °C; (**c**) 30 °C; (**d**) 40 °C; (**e**) 50 °C; (**f**) 60 °C.

Figure 3 shows the typical failure modes for the test specimens, which exhibit different patterns after exposure at variable temperatures and pre-sustained loading conditions. The failure mode of the control specimen was primarily cohesion failure at the thin concrete layer (Figure 3a); this is the typical failure mode for the FRP-concrete shear pull-off test [15]. For the specimens tested at 15 and 30 °C with pre-sustained loading (Figure 3b,c), the failure mode is the same as that of the control specimen implying that the failure modes below 40 °C are controlled by concrete tensile strength. It was reported that the pre-sustained load with 23 °C may increase the failure area of the interfacial debonding [11]. That phenomenon is different from the present results below 40 °C and is attributed to the duration of the pre-sustained load (251 days) in [11]. Compared to the present duration of pre-sustained loading, longer durations accumulate the damage at the interface between adhesive layer and the concrete.

In the case of an environment temperature at 40 °C or higher, the failure mode changes from concrete failure to adhesion failure at the adhesive-to-concrete interface. Figure 3d–f shows that the complete debonding between the adhesive layer and concrete occurs for the specimens from 40 to 60 °C. *T*_g_ in the adhesive layer dominates the failure modes above 40 °C in the present cases. The environment temperature between 40 and 60 °C deteriorates the primer–concrete interfacial bond. It was reported that the temperature above *T*_g_ changes the failure mode from concrete cohesive failure to interfacial debonding [15,33]. The adhesion between adhesive layer and concrete has higher resistance than that of concrete to instantaneous loading [23], which indicates that the failure evolution depends on the temperature rather than the pre-sustained load within a short duration (e.g., 3 h).

The previous studies were reported that the weakest region occurs at the surface of the concrete substrate at the temperature below *T*_g_ [8,15,34]. The fracture energy of the failure in concrete is smaller than that of the failure at the primer–concrete interface. It was reported that the value of interfacial fracture energy was approximately 0.04 N/mm for the concrete, while the interfacial fracture energy between FRP and concrete is more than 3 N/mm [35]. In the case of EST, the change of the failure mode occurs when the temperature exceeds the *T*_g_. This indicates that the fracture energy of the interfacial bond between primer and concrete rapidly decreases when the temperature is above *T*_g_. However, in the present case at 40 °C, which is below the *T*_g_, the failure mode has shifted from concrete cohesive failure to adhesive layer-concrete debonding. Ferrier et al. results were reported that at 60 °C without a pre-sustained load, the failure mode of the CFRP sheet-concrete with *T*_g_ = 76 °C also shifted to interfacial debonding [12]. This indicates that a temperature close to *T*_g_ may shift the failure mode from concrete cohesive failure to interfacial debonding. It is difficult to distinguish the effect of the pre-sustained loading with 35% of failure load on the change of failure mode at 40 °C in present study. The effects of pre-sustained load on the failure mode at temperatures close to *T*_g_ should be further investigated.

### 3.2. Load-Slip of BFRP–Concrete Bond

**Figure 4:**
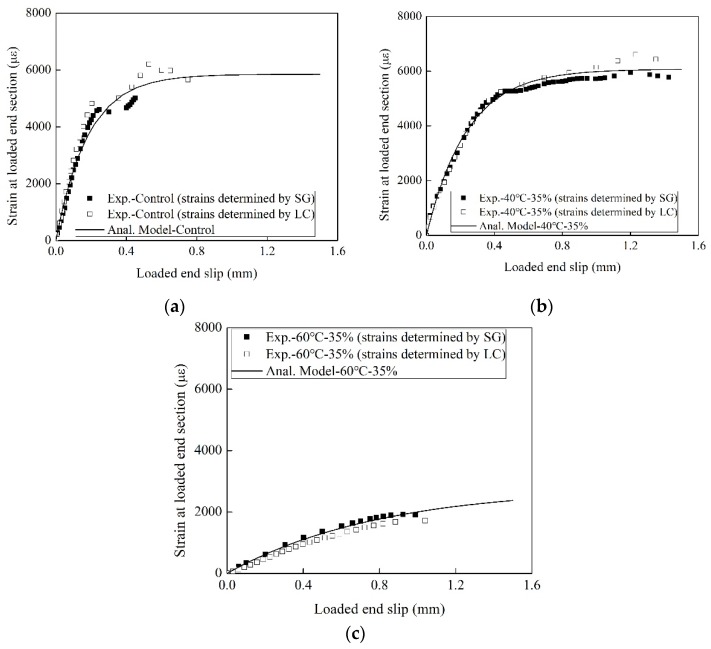
The typical measured strain–slip data and their corresponding regressed fitting curve at: (**a**) 15 °C; (**b**) 40 °C; (**c**) 60 °C.

Figure 4 shows the comparisons of strain–slip curves. “Exp.-X°C-35%” represents the strain read by the strain gauge on the FRP. “Exp. Results determined by load” indicates the strain determined by load. “Ana.-X°C-35%” is determined according to Dai et al. equation [29]. The strain–slip curve of “Exp.-X°C-35%” matches that of “Exp. Results determined by load” in Figure 4. 

Dai et al.’s equation was adopted to evaluate the BFRP-to-concrete bond performance. The interfacial bond stress (*τ*) is expressed as follows [29]:

(2) $$\tau =A^{2}BE_{f}t_{f}\left(e^{-Bs}-e^{-2Bs}\right)$$

where *τ* is a function of slip *s*.

During the pull-out test, the pull-out forces, strain of BFRP, and the slip at the loaded end can be measured accurately through a load cell, strain gauge, and displacement transducer. As a result, the relationship between the strains of BFRP sheets and the slips at the loaded end, in other words, the function of *f*(s), can be obtained directly from simple pull-out tests.

Strain is assumed to be related to slip:

(3) $$f\left(s\right)=\varepsilon =A\left(1-e^{-Bs}\right)$$

where *A* and *B* can be fitted by the *ε-s* curve of the experimental results. In fact, *A* is the maximum strain in the FRP with a sufficiently long bond length. *B* is regarded as the stiffness index that controls the shape of the bond-slip curve [36]. *A* and *B* for all the specimens were obtained by fitting Equation 3 and are listed in Table 1. 

The ultimate load capacity is determined by the maximum strain at the loaded end as follows:

(4) $$P_{u}=E_{f}t_{f}b_{f}\varepsilon_{\max}$$

where *b*_f_ is the width of the BFRP. It is worth noting that *ε*_max_ is equal to *A*. The evolution of the elastic modulus of the BFRP sheet at temperatures from 30 to 60 °C were determined by the modified Bisby’s model [9,37]. *P*_u_ values for all specimens are listed in Table 1.

Figure 4 shows the typical measured strain–slip data and the corresponding regressed fitting curve at 15, 40, and 60 °C. In comparisons of CT15 and T15, the pre-sustained load increased *A* and *B* by 1.4% and 13%, respectively. Compared to the cases at 15 °C with pre-sustained loading, the values of *A* at 30 and 40 °C increase by 20% and 9%, respectively. The temperature below *T_g_* enhances the hydrogen bonds between the epoxy and SiO_2_ (contained in concrete). The value of *B* for both at 15 and 30 °C are similar. Compared to the specimens at 15 °C, the temperature of 40 °C reduces the value of *B* by 58%, which is attributed to the deterioration of the primer–concrete interfacial bond. 

Compared to the specimens at 15 °C, the EST of 50 and 60 °C reduces the values of *A* by 38% and 44%, respectively, due to the deterioration of the primer–concrete bond. The value of *A* is controlled by the primer–concrete interfacial bond, which decreases with temperature. Compared to the specimens at 15 °C, the values of B at 50 and 60 °C decrease by 73% and 82%, respectively, attributed to the softening of the bonding adhesive, and the disruption of bonds between the primer and concrete.

### 3.3. Shear Bond Strength and Fracture Energy

According to references [29], the bond stress–slip relationship and fracture energy can be expressed as:

(5) $$\tau =A^{2}BE_{f}t_{f}\left(e^{-Bs}-e^{-2Bs}\right)$$

(6) $$G_{f}=\frac{1}{2}A^{2}E_{f}t_{f}$$

**Figure 5:**
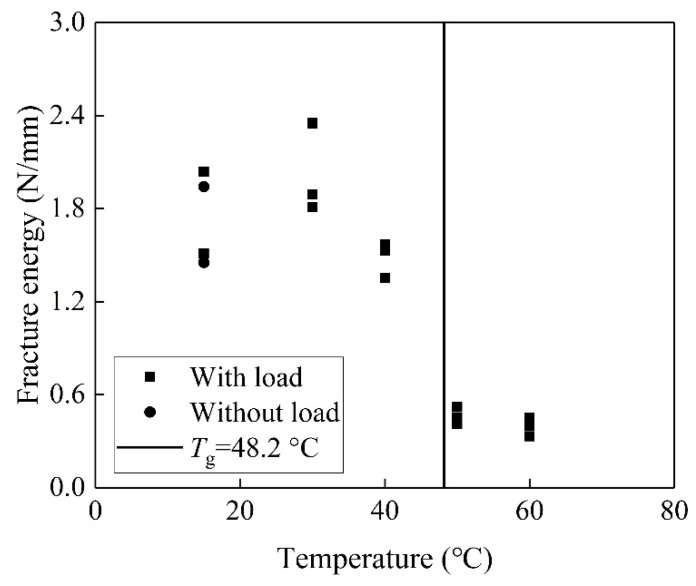
Interfacial fracture energy vs. temperature at the surface of BFRP.

The fracture energy is determined by Equation 6 and listed in Table 1. Figure 5 shows an insignificant effect of pre-sustained loading on the fracture energy at 15 °C. Compared to the case at 15 °C, the fracture energy at 30 °C increases by 46%. The temperature results in post-curing and stronger bonds between the primer and concrete.

Compared to the cases at 15 °C, the fracture energy at 40 °C only increases by 11%, which is lower than that at 30 °C. It is worth noting that the trends of fracture energy at 40 °C are different from those of the values of *A* and *B*. It was reported that the environment temperature below *T*_g_ influences the fracture energy insignificantly [38]. The pre-sustained loading at 40 °C accelerates the debonding between the primer and concrete. Meanwhile, a temperature of 40 °C enhances the crosslinking of the epoxy and primer-SiO_2_ interfacial bond, thus resulting in a higher value of *A* and a lower value of *B* at 40 °C.

The fracture energy decreases rapidly when the temperature at the surface of BFRP reaches 50 °C, which is above *T*_g_. Compared to the specimens at 15 °C, the fracture energies at 50 and 60 °C reduce by 73% and 77%, respectively. The mobility of the chain in the epoxy increases with EST. The epoxy is rather soft and exhibits a large deformation above 50 °C, which reduces the shear stiffness of the bond layer involved in a combined layer of primer and adhesive. It is reported that the fracture energy increases with the decreasing shear stiffness of the bond layer [29]. In fact, the fracture energies of the specimens at both 50 and 60 °C are smaller than that at 15 °C. It is indicated that the pre-sustained loading and EST significantly deteriorates the bond between the primer and concrete.

**Figure 6:**
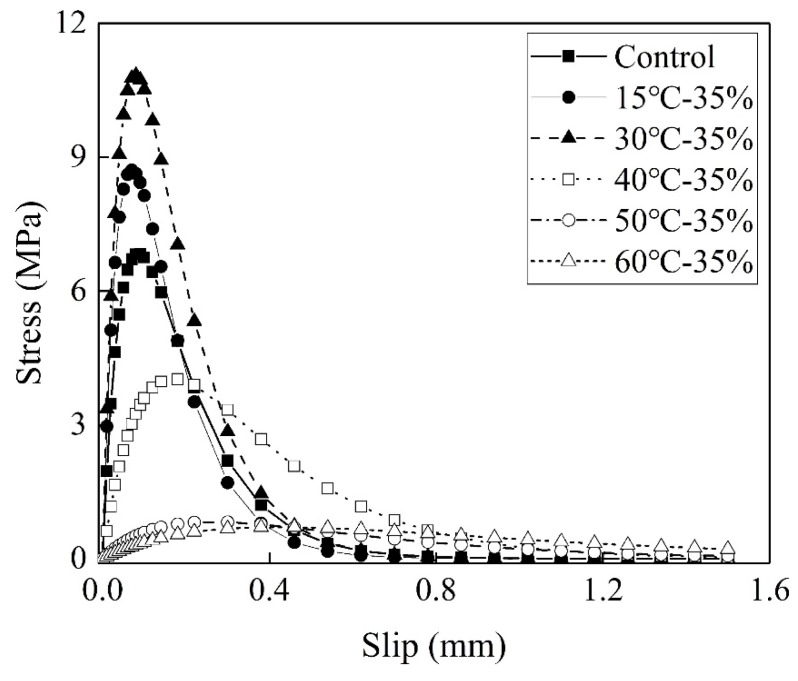
Bond stress–slip relationship.

The bond stress–slip relationship determined by Equation 5 is plotted in Figure 6. According to Ref. [29], the maximum shear bond stress and the corresponding slip can be expressed as:

(7) $$s_{0}=\frac{\ln2}{B}$$

(8) $$\tau_{\max}=0.5BG_{f}$$

At 15 °C, the pre-sustained load increases the shear bond stress by 18.6%. As discussed above, the pre-sustained load increases *B* by 13%. According to Equation 8, the increase in the shear bond stress appears to result from the increase in *B*. 

The shear bond stress increases with increasing environment temperature from 15 to 30 °C. This is attributed to the stronger interfacial bond owing to the post-curing. The shear bond stress decreases with the environment temperature above 40 °C, while the corresponding slip increases. The corresponding slip decreases owing to the decrease in *B*. The rapid decrease in the shear bond stress at 40 °C is attributed primarily to the shift in failure mode from concrete cohesive failure to interface debonding. 

The maximum shear bond stress of the CFRP sheet-concrete with *T*_g_ =76 °C of adhesive epoxy increased by 18% at temperature from 20 to 40 °C [12]. In the present study, the maximum shear bond stress increased 23% at the temperature from 15 to 30 °C. The trends of maximum shear bond stress with temperature are similar for both the present study and the CFRP sheet-concrete in [12]. The difference in the increase between 18% and 23% is insignificant. 

After 153 days of pre-sustained loading at 45% of the failure load the maximum shear bond stress of CFRP sheet-concrete increased by 65% [11]. It can be concluded that the maximum shear bond stress of the CFRP sheet-concrete increased with temperatures. The pre-sustained load below 45% of the failure load has positive effects on the maximum shear bond stress of CFRP sheet-concrete, and it results in the increase of the maximum shear bond stress with loading duration.

The temperature of 60 and 80 °C reduced the maximum shear bond stress of the CFRP sheet-concrete by 31% and 66%, respectively [12]. In the present study, 58% and 93% reductions in the maximum shear bond stress of the CFRP sheet-concrete for the temperature of 40 and 60 °C were found, respectively. The coupling of pre-sustained load and EST deteriorated the maximum shear bond stress more severely than did elevated temperature conditions. As discussed above, the positive effects of pre-sustained load below 50% of the failure load on the maximum shear bond stress are evident. It is concluded that the 35% of the failure load accelerates the deterioration of the maximum shear bond stress.

### 3.4. Effective Bond Length

**Figure 7:**
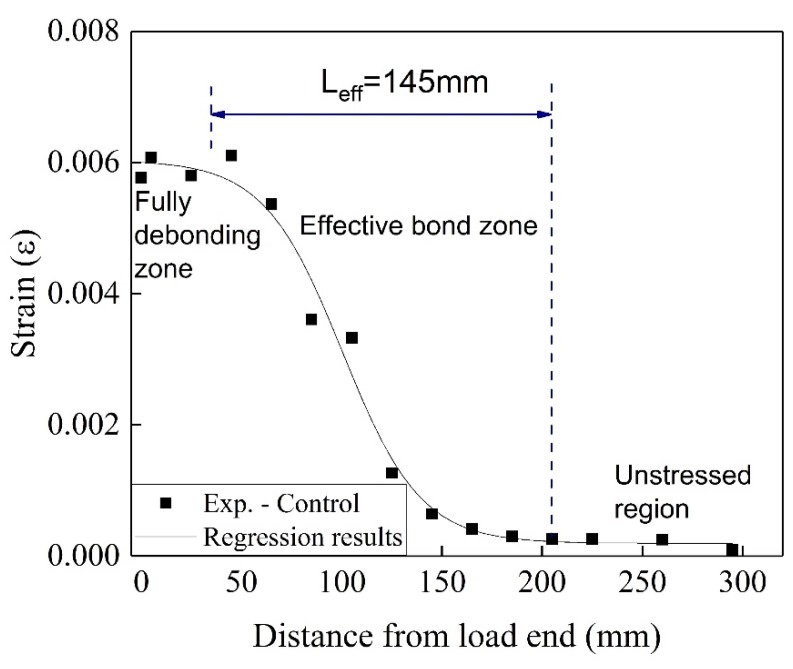
Effective bond length for the reference specimen.

The strains on the surface of the CFRP plate were measured by strain gauges placed at intervals of 20 mm. The observed strain distribution can be divided into three regions: (1) The unstressed region near the free end where the strain reaches the peak strain; (2) the effective bond zone, which is the distance from the end of peaking strain to the beginning of strain plateau (approximately zero); (3) the fully debonding zone where the strain plateaus (Figure. 7). 

The strain distribution as a function of the distance from the free end can be subjected to regression analysis using the following expression [39]:

(9) $$\varepsilon \left(y\right)=\varepsilon_{0}+\frac{\alpha }{1+e^{\left(\frac{y-y_{0}}{\beta }\right)}}$$

where *ε*_0_, *α*, *y*_0_, and *β* are constants determined by nonlinear regression analysis. After conducting the regression analysis, the strain distribution at the peak loads calculated using Equation 9 is plotted (solid line in Figure 7). The effective bond lengths (*L*_eff_), representing the experimental results, as determined by Eq. 9, are listed in Table 1.

When the bonded length is long enough, the effective bond length is also predicted as a function of *L*_e_, *B,* and *G*_f_ [40].

(10) $$L_{e}=\frac{\sqrt{2E_{f}t_{f}}}{B\sqrt{G_{f}}}\ln(\frac{1+\alpha }{1-\alpha })$$

Where *α* is regarded as a constant percentage for anchorage design. The percentage *α* = 0.96 was fitted with experimentally observed effective bond length well [41] and is the value assumed in the present study. 

**Figure 8:**
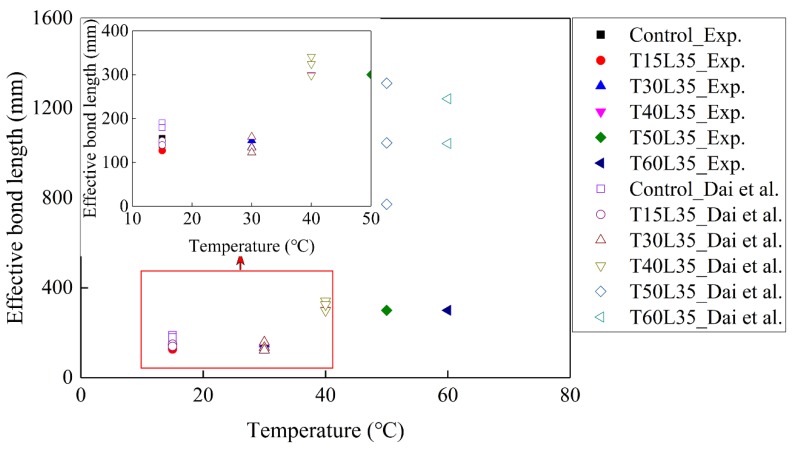
Comparisons of predicted and tested results.

Figure 8 shows the comparison of effective bond length determined by Equations 9 and 10, respectively. The effective bond length between 15 and 40 °C determined by Equation 10 is similar to the test results, which indicates that Equation 10 can accurately predict the effective bond length. The differences in effective bond length determined by Equations 9 and 10 are significant for the case of 50 and 60 °C meaning that Equation 10 is unable to obtain the effective bond length in the case of insufficient bond length. In the case of 50 and 60 °C, Figure 8 shows that the effective bond length determined by Equation 10 is larger than that of the actual bond length (larger than 300 mm).

Figure 8 shows that the effective bond length did not vary from 15 to 30 °C. The effective bond length is related to the stiffness of the FRP and the concrete compressive strength according to Equation 1. The stiffness of the FRP and the concrete compressive strength remained stable within a range from 15 to 30 °C [15]. It was assumed that the elastic modulus of BFRP from 15 to 30 °C below *T*_g_, was not changed in present study. In comparisons of the control specimens at 15 °C, the effective bond lengths of the specimens with three hours of pre-sustained loading from 15 to 30 °C are similar, indicating that the short-pre-sustained load duration (three hours) insignificantly influenced the effective bond length when the temperature was below *T*_g_.

**Figure 9:**
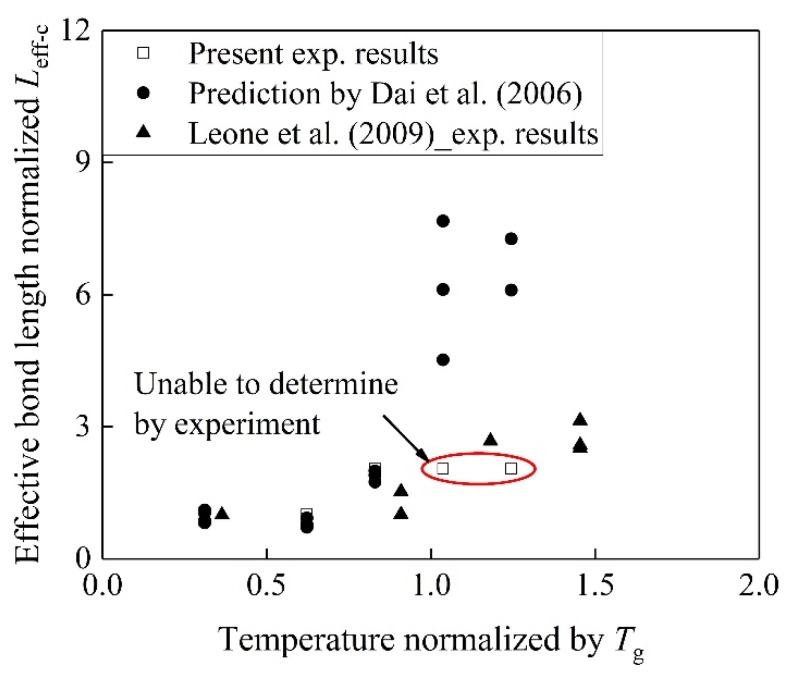
Effective bond length normalized by *L*_eff-c_ vs. temperature at surface of BFRP normalized by *T*_g_.

It is worth noting that the effective bond lengths for the specimens at 40, 50, and 60 °C are more than 300 mm in Figure 8. The previous study showed that in the case of *T*/*T*_g_ =0.9, the effective bond length did not vary. In the present study, 40 °C is *T*/*T*_g_ = 0.82, which is smaller than *T*/*T*_g_ = 0.9, and the effective bond length is larger than 300 mm. Figure 9 shows the comparisons of the present results, predicted by Equation 10 from Dai et al. [40] and those cited from Leone et al. [8]. “*L*_eff-c_” means the effective bond length of the control specimens. The effects of the variation temperature on the bond lengths were considered by Leone et al. [8]. This means that the effective bond length with *T*/*T*_g_ = 0.82 is at least two times larger than that of the control specimens, which is attributed to the pre-sustained load at 40 °C that causes the rapid increase of the effective bond length. The detailed values of the effective bond lengths at 40, 50, and 60 °C are difficult to determine by Equation 9. In the present study, the effective bond lengths with coupling of *T*/*T*_g_ = 1.04 or 1.24, and pre-sustained load can be determined by Eq. 10, and were found to be 1.8 and 2.5 times larger than that of the control specimens, respectively. It was reported that *T*/*T*_g_ = 1.50 increases the effective bond length by 260% [8] suggesting that the effects of EST on the effective bond length are significant. The pre-sustained load enlarges the effective bond length, and increases the effective bond length with EST. 

The combined effects of EST and pre-sustained load on the effective bond length can be explained as follows. First, the low elastic modulus of epoxy at EST results in the longer effective bond length. Next, the redistribution of shear stress causes the pre-sustained loading along the FRP due to deterioration of the primer–concrete interfacial bond.

## 4. Degradation Mechanism

**Figure 10:**
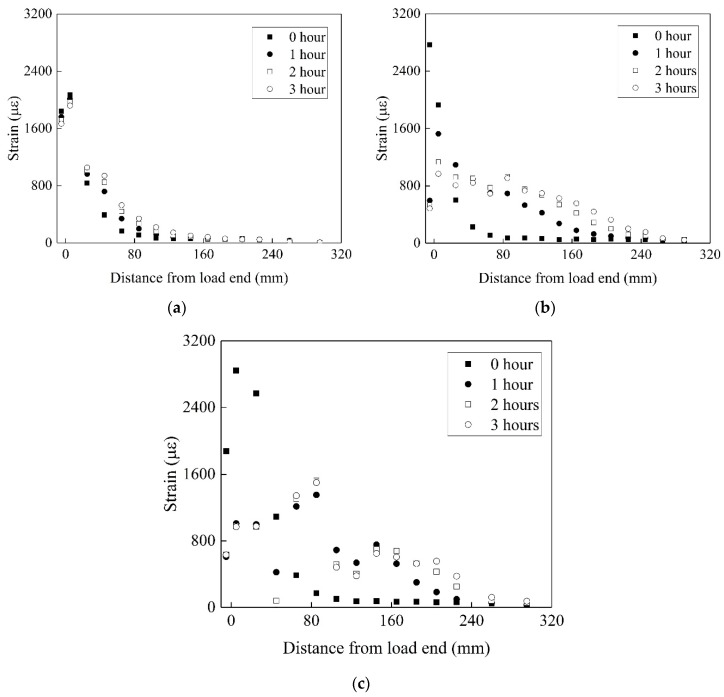
Strain vs. distance from load end: (**a**) 15 °C, (**b**) 40 °C, (**c**) 50 °C.

Figure 10 shows the strain gauge reading at various times of pre-sustained loading with 35% of the ultimate load capacity. The exponential trend extends over the bond length after the load application. Because of the extremely high shear stresses at the adhesive and concrete cover level, the creep phenomenon is highly nonlinear close to the loaded end.

A longer portion of the bond length is subjected to shear stress transfer within the duration of pre-sustained loading and temperature. The distance required for the strain to reach zero at 15 and 30 °C is 85 mm at 0 h in both cases. The load transfer zone for 15 and 30 °C reaches 145 and 185 mm after 3 h, respectively. This is attributed primarily to the creep of the primer–concrete interfacial bond [42], and results in the redistribution of shear stress between the primer and concrete along the FRP [43]. 

An increase in temperature accelerates the interfacial creep between the primer and concrete. The load transfer zone increases with temperatures above *T*_g_. Compared to the case at 30 °C, the load transfer zone increases by 362% for 50 °C. First, the increase in temperature results in the deterioration in the epoxy properties (Young modulus) and then, EST increases the primer–concrete interfacial slip [12]. 

At the stage of ST, the strain at the loaded end rarely varies at 15 °C. The strain reduces by 7.4% after three hours of pre-sustained load. The amplitude of the decrease in the strain at the loaded end increases with temperature. After three hours of pre-sustained load, 30 and 40 °C reduces the strain by 25% and 50%, respectively, while 60% and 54% reductions in strain were found for 50 and 60 °C, respectively. It appears that the effects of the temperature from 40 to 60 °C on the reduction of strain are similar. 

An obvious reduction in the strain gradient near the loaded end occurs from 40 to 60 °C, indicating the onset of stable debonding between the primer and concrete near the loaded end [11]. It is worth noting that the strain near the bonded end reduces, although 40 °C is lower than *T*_g_. This implies that interfacial debonding and creep occur prematurely because of the pre-sustained loading.

**Figure 11:**
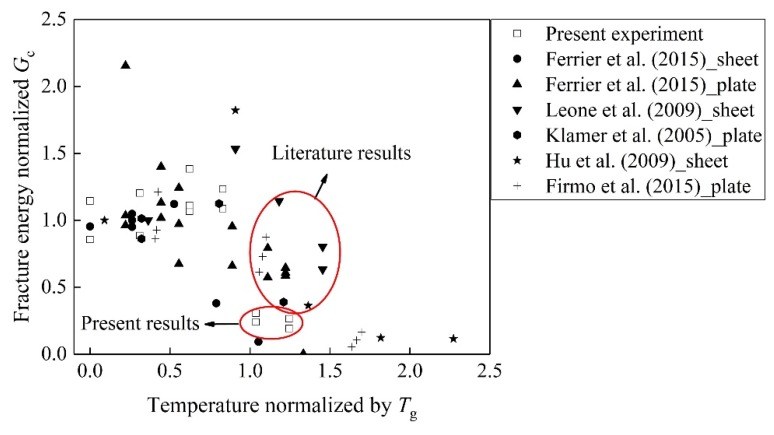
Fracture energy normalized by *G*c vs. temperature at surface of BFRP normalized by *T*_g_.

To distinguish the effects of the variable temperature and pre-sustained load on the bond, the previous studies [8,15,33,44,45] were introduced. In all of the studies only the effects of temperature variation were taken into account. Figure 11 shows the comparisons of the present results and the previous results. “*G*c” means the interfacial fracture energy of the control specimens. In the range *T*/*T*_g_ between 0 and 1, the pre-sustained load influences the interfacial fracture energy insignificantly. The temperature linearly increases the interfacial fracture below *T*_g_ in Figure 11. When *T*/*T*_g_ exceeds 1, the reduction in fracture energy in present study is larger than that in the literature results indicating that the pre-sustained load plays an important role in the degradation of the interface bond. 

**Figure 12:**
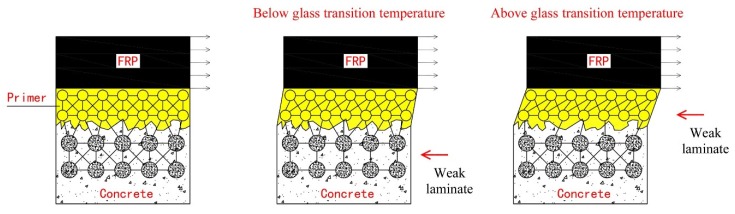
Schematic illustration of the degradation mechanism.

Figure 12 shows the schematic illustration of the degradation mechanism. “Weak laminate” in Figure 12 denotes the primary damaged laminate. Figure 12 (the middle photo) illustrates that the failure mode is dominated by the concrete tensile strength for the cases of 15 and 30 °C. The positive effects of epoxy post-curing results in an increase in the interfacial fracture energy. The adhesive creep and interfacial bond are insignificant to the fracture energy. 

The failure mode shifts from concrete failure to primer–concrete interfacial debonding as the exposure temperatures exceeds 40 °C. Figure 12 (the right photo) shows the primer–concrete interfacial bond that involves the physical bonding and interlocking interaction. The coupled effects of variable elevated temperature and pre-sustained loading on the interfacial bond are significant. First, the temperature close to *T*_g_ and above disrupts the physical bond between the primer and concrete [46]. The pre-sustained loading results in the creep of the interfacial bond, and accelerates the disruption of the physical bond between the primer and SiO_2_ [47]. Next, the softened primer can be easily removed from the concrete surface under the pre-sustained loading owing to its low elastic modulus, thus the interlocking interaction decreases. 

**Figure 13:**
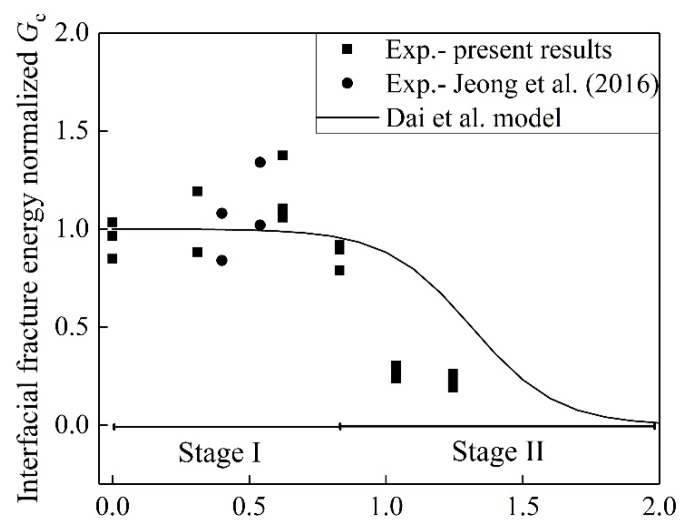
Effects of the pre-sustained load on the FRP-to-concrete bond.

Figure 13 shows the coupled effects of temperature variation and pre-sustained loading on the FRP-to-concrete bond. The model of Dai et al. was adopted to determine the effects of variable temperature on interfacial fracture energy [9]. “Temperature normalized by *T*_g_” was defined as the ratio of the temperature at the surface of BFRP to the glass transition temperature. The temperature normalized by Tg between 0 and 2 can be divided into two stages: Stage I and Stage II, as shown in Figure 13. In the case of Jeong et al. (2016), CFRP sheet was used. Compared to the present study, it is worth noting that the longer pre-sustained load duration of 79 and 153 days were applied for the specimens with temperature normalized *T*_g_ = 0.54 and 0.4, respectively. As shown in Figure 13, the longer duration insignificantly influenced the bond strength at Stage I. It was reported that less than 50% of the ultimate bond strength involved no-creep bond failure [23]. It is interesting that the normalized fracture energy increases until the temperature normalized *T*_g_ reaches 0.83. In comparison with the fracture energy determined by the Dai et al. model, Stage I shows that the fracture energy from the experiment is larger below 40 °C. This is attributed to the positive influence of temperature on the interfacial bond. The Dai et al. model ignores the effects of temperature variation on the epoxy because of the post-curing during Stage I. 

The interfacial fracture energy for cases above 50 °C is smaller than the results determined by the Dai et al. model. Compared to the prediction of the Dai et al. model, the pre-sustained loading reduces the fracture energy by 62% and 69% for 50 and 60 °C, respectively. This implies that the pre-sustained loading accelerates the deterioration in the interfacial fracture energy. 

## 5. Conclusions

The coupled effects of variation temperature and pre-sustained loading on the bond behavior between BFRP sheets and concrete were studied both experimentally and analytically. Based on the results and discussion, the conclusions are as follows: 

(1) Within the pre-sustained loading (35% of *f*_u_), the failure mode is primarily cohesion failure in the thin concrete layer at the temperature of 15 and 30 °C. However, the failure mode is changed to primer–concrete interface debonding at the temperature of 40, 50, and 60 °C due to the decrease of the physical bonding and interlocking interaction.

(2) Effects of pre-sustained loading (35% of *f*_u_) on the failure load at temperature 15 and 30 °C is insignificant. The coupling of the temperature (i.e., 40, 50, and 60 °C) and pre-sustained loading reduced the failure load by 21%, 56%, and 59%, respectively. 

(3) Compared to the temperature at 15 °C, the fracture energy increases by 46% at 30 °C and 11% at 40 °C. However, it reduces by 73% and 77% at 50 and 60 °C, respectively. The effect of the pre-sustained loading has the insignificant influence on the fracture energy at the temperature of 15 and 30 °C. The pre-sustained loading accelerated the degradation of the interfacial bond at the temperature above 30 °C. 

(4) The short duration of pre-sustained load (three hours) insignificantly influenced the effective bond length at the temperature of 15 and 30 °C. The effective bond length at the temperature above 30 °C was at least two times larger than that of the control specimens. The effective bond length increased with the temperature. 

(5) After three hours of the steady-state test (ST), the strain at the loaded end decreased with increasing temperature. The reduction in strain at the loaded end maintained a stable value at the temperature of 40, 50, and 60 °C.

## Figures and Tables

**Table 1:** Details of specimens and pullout bond test results.

Specimens No.	Load (0.35*f*_u_)	Temperature (°C)	*A* (με)	*B* (mm^−1^)	τ_max_ (MPa)	*S_0_* (mm)	*P_u_* (kN)	*G_f_* (N/mm)	*L_e_* (mm)	Failure Modes
CT15-1	No	15	5850	7.6	6.71	0.08	33.20	1.65	145	C^1^
CT15-2	No	15	5593	9.7	8.6	0.07	31.72	1.77	140	C^1^
CT15-3	No	15	5059	8.6	6.24	0.07	28.69	1.45	155	C^1^
T15L35-1	Yes	15	6001	8.7	8.92	0.07	34.04	2.04	127	C^1^
T15L35-2	Yes	15	5150	10.8	8.12	0.06	29.21	1.51	140	C^1^
T15L35-3	Yes	15	-	-	-	-	-	-	-	C^1^
T30L35-1	Yes	30	6150	8.0	7.24	0.08	29.44	1.81	122	C^1^
T30L35-2	Yes	30	7003	8.3	9.74	0.08	33.53	2.35	150	C^1^
T30L35-3	Yes	30	6276	10.1	9.52	0.06	30.05	1.89	135	C^1^
T40L35-1	Yes	40	6075	4.3	3.29	0.15	25.23	1.53	300	I^2^
T40L35-2	Yes	40	5703	4.0	2.70	0.16	23.69	1.35	300	I^2^
T40L35-3	Yes	40	6142	3.9	3.06	0.16	25.51	1.57	300	I^2^
T50L35-1	Yes	50	3536	2.1	0.54	0.30	14.62	0.52	300	I^2^
T50L35-2	Yes	50	3136	3.2	0.65	0.20	12.97	0.41	300	I^2^
T50L35-3	Yes	50	3294	1.8	0.40	0.36	13.62	0.45	300	I^2^
T60L35-1	Yes	60	3290	1.9	0.43	0.34	13.60	0.45	300	I^2^
T60L35-2	Yes	60	2806	1.4	0.23	0.46	11.60	0.33	300	I^2^
T60L35-3	Yes	60	3116	2.4	0.47	0.27	12.88	0.40	300	I^2^

^1^ The concrete cohesive failure; ^2^ the debonding between adhesive layer and concrete.
